# L-Arabinose Alleviates Functional Constipation in Mice by Regulating Gut Microbiota and Metabolites

**DOI:** 10.3390/foods14050900

**Published:** 2025-03-06

**Authors:** Ke Liu, Huixuan Dong, Xinran Li, Chaoqun Hu, Fengya Cui, Shiji Li, Xiaolin Zhang, Yushan Du, Penghui Yang, Wenna Ji, Wenjie Sui, Jing Meng

**Affiliations:** 1 State Key Laboratory of Food Nutrition and Safety, College of Food Science and Engineering, Tianjin University of Science and Technology, Tianjin 300457, China; liukegj@163.com (K.L.); a741846041@163.com (H.D.); lixrrr@163.com (X.L.); cuifengya666@163.com (F.C.); lishiji1024@163.com (S.L.); zz9951011@163.com (X.Z.); 18232849193@163.com (Y.D.); vnbzs54683@163.com (P.Y.); wjsui@tust.edu.cn (W.S.); 2 Healtang Biotech Co., Ltd., Zhangqiu District, Jinan 250204, China; huchaoqun@healtang.com (C.H.); jiwenna@shengquan.com (W.J.); 3 Jinan Fruit Research Institute, All-China Federation of Supply & Marketing Co-Operatives, Jinan 250014, China

**Keywords:** functional constipation, L-Arabinose, gut microbiota, metabolites

## Abstract

Functional constipation ranks among the most common disorders impacting human health, which is manifested by difficulty in defecation and a complex etiology. L-Arabinose, a pentose found naturally in fruit rinds and cereal husks, has been reported to regulate glycolipid metabolism, improve glucose homeostasis, and exhibit anti-inflammatory effects. However, the effect and precise mechanism of L-Arabinose on functional constipation remain unclear. In this study, the effect of L-Arabinose in alleviating functional constipation induced by diphenoxylate was evaluated. The model group consisted of functional constipation mice that did not receive any intervention. The positive drug group was treated with 2.0 g/kg lactulose, while the intervention group was given 0.5 g/kg, 0.75 g/kg, 1.0 g/kg, and 2.0 g/kg L-Arabinose, respectively. The data suggested that 20 days of L-Arabinose intervention could shorten the first black stool defecation time, increase fecal water content, and enhance the rate of small intestinal propulsion in mice with functional constipation induced by diphenoxylate. Additionally, L-Arabinose reversed the protein expression of functional constipation-related intestinal factors in the colon, characterized by a decrease in the expression of water channel proteins AQP3 and AQP4, as well as an increase in the expression of tight-junction proteins ZO-1, Claudin-1 and Occludin. Furthermore, L-Arabinose modulated the levels of hormones (MTL, Gas) and neurotransmitters (5-HT, VIP) related to the digestive systems of mice with constipation, resulting in elevated levels of 5-HT, MTL, and Gas and decreasing levels of VIP. Histopathological analysis also revealed that L-Arabinose intervention improved the intestinal inflammatory response. Furthermore, 16S rRNA sequencing and metabolomics of the intestinal microbiota demonstrated that L-Arabinose treatment improved both the intestinal microbiota composition and the metabolite levels. This study suggests that L-Arabinose can serve as a potential functional ingredient to promote intestinal health, enhance gastrointestinal motility and barrier function, regulate osmotic pressure, restore neurotransmitter levels, and effectively relieve functional constipation.

## 1. Introduction

The prevalence of constipation is rising annually due to societal aging, increased stress levels in modern life, and shifts in dietary habits and lifestyle pace. Worldwide, constipation is believed to affect 2–27% of the population [1]. Constipation symptoms have various effects on daily life, significantly decreasing the physical and mental health-related quality of life [2]. Constipation can be classified into two primary categories based on its pathogenesis: organic constipation and functional constipation. Organic constipation is primarily attributable to lesions in the gastrointestinal organs, whereas functional constipation represents a prevalent clinical subtype characterized by a high incidence of occurrence [3]. It is characterized by lumpy or hard stools, infrequent bowel movements, abdominal cramps, and bloating [4]. Prolonged constipation can lead to physical anal tears, hemorrhoids, and other undesirable conditions [5]. Additionally, severe constipation may result in dangerous physical symptoms such as fainting and may predispose individuals to the development of rectal cancer [6], colon cancer, and cardiovascular disease [7]. In recent years, an increasing number of studies have recognized that the pathogenesis of constipation is highly complex, involving multiple factors throughout the gastrointestinal tract, such as gastrointestinal motility, hormone secretion, microecological balance, metabolite alterations, and barrier function [8,9]. The etiology of constipation is varied, encompassing insufficient hydration/fiber consumption, and aberrant bowel function, decreased physical activity, and underlying medical conditions [10,11]. Therefore, a thorough analysis linking gut health and bowel irregularity is key for developing more effective interventions aimed at preventing and alleviating constipation symptoms [12]. Extensive research highlights that probiotics have multifaceted advantages and various potential benefits for the relief and treatment of constipation. Probiotics modify the gut microbiota among constipated individuals [13], leading to a normalization of intestinal microorganisms. Probiotics elevate intestinal lactate and short-chain fatty acid concentrations, which further reduce the pH value [14], promote intestinal peristalsis, and decrease the intestinal transit time, thereby improving constipation symptoms. Probiotic byproducts significantly modulate gut activity, including neurotransmission and motility [15]. Furthermore, research indicates that some plant-derived polysaccharides offer relief from constipation [16]. For instance, konjac glucomannan can enhance intestinal motility by increasing the rate of small intestinal propulsion [17], while chrysanthemum polysaccharides can improve the structure of the intestinal flora in functionally constipated rats [18], leading to increased species abundance and diversity. Candida polysaccharides can improve constipation in rats by increasing fecal wet weight [19]. *Cistanche deserticola* polysaccharides can regulate the microbial richness and diversity in constipated rats [20]. In clinical practice, commonly used medications for constipation include bulking laxatives and secretory laxatives [21]. However, these therapies frequently induce adverse reactions, including distension, stomach discomfort, and sickness, and may not be well tolerated by many patients. Therefore, a non-dependent and safe strategy for treating constipation is a promising choice.

L-Arabinose, a pentose unique to plants, is abundant in the environment, obtainable from wheat bran, beet pulp, or corn stalks [22]. L-Arabinose exists in two forms, L-arabinofuranose (L-Ara*f*) and L-arabinopyranose (L-Ara*p*), and is an important component of many polymers [23]. Free L-Arabinose exists in solution as L-Ara*p* because the pyranose form is more stable than the furanose [24]. Meanwhile, L-Arabinose is one of the components of hemicellulose and pectin polymers [25]. Its structural characteristics make it play an important role in the composition of plant cell walls. L-Arabinose, in vitro, curtails maltase and sucrase function within the intestine [26]. In rats, the consumption of dietary sucrose elevates insulin levels in the blood and triacylglycerol levels in the plasma and liver. However, the concurrent feeding of L-Arabinose with sucrose significantly mitigates the rise in these levels [27]. L-Arabinose exhibits anti-inflammatory properties [28], relieves lipid metabolism disorders [29], reduces cholesterol levels, and improves dyslipidemia [30]. Furthermore, it has been shown to alleviate colitis by influencing the microbial composition of the gut [31]. Compared to other prebiotics, L-Arabinose uniquely inhibits sucrose absorption, thereby offering dual benefits in blood glucose regulation and constipation relief. As a common constituent of various plant polysaccharide hemicelluloses, L-Arabinose has a wider range of sources and more diverse production methods. Presently, research on the potential of L-Arabinose in alleviating functional constipation remains limited. Most existing studies on L-Arabinose have focused largely on its effects on the gut microbiota [32], but this paper extends the investigation to explore metabolites and the intricate relationships between the gut microbiota and metabolites.

Diphenoxylate created a murine analog of functional constipation for this investigation. Subsequently, L-Arabinose’s role and action in mitigating constipation were examined holistically by examining fecal water content, small intestine propulsion, osmotic pressure balance, intestinal barrier integrity, gut microbiota and its metabolites. This analysis aimed to provide a theoretical foundation for the application of L-Arabinose in improving functional constipation.

## 2. Materials and Methods

### 2.1. Animals and Experimental Design

We obtained KM mice (4–6 weeks old, weighing 18–20 g) from SPF Biotechnology Co., Ltd., (Beijing, China). After acclimatization and a 3-day feeding period in the animal house, the mice were randomized using the random number table method and divided into the control group (C, *n* = 10) and the experimental group (*n* = 60). Mice in the control group received 0.9% saline by oral gavage, while the mice in the modeling group were administered compound diphenoxylate tablets by gavage (Henan Dingchang Pharmaceutical Co., Ltd., Xinxiang, China). Diphenoxylate was administered once daily for 7 days to establish a constipation model. To further investigate the effects of different doses of L-Arabinose on constipation, the mice that underwent the 7-day modeling were then randomly re-divided into six groups: the model group (M), the positive control group (R), and four L-Arabinose dosage administration groups (A1–A4), again using the random number table method. During the 20-day administration period, the mice in the C and M groups were given 0.9% saline. The mice in group R received 2.0 g/kg of lactulose (J&K Scientific Ltd., Beijing, China). The mice in groups A1–A4 were, respectively, administered 0.5 g/kg, 0.75 g/kg, 1.0 g/kg, and 2.0 g/kg of L-Arabinose A40 (Healtang Biotech Co., Ltd., Jinan, China) by gavage once daily for 20 days.

To evaluate the experimental outcomes, feces were collected every other day to determine the water content, and the first black stool defecation time was measured every three days. The effectiveness of the modeling was assessed based on differences in fecal characteristics, fecal water content, and the first black stool defecation time after modeling. To minimize potential bias, a double-blind method was employed throughout the experiment. Different researchers participated in the experimental operation, data collection, and result evaluation stages and blinded the mouse grouping.

### 2.2. Water Content in Faeces

Mouse faeces were collected to determine their water content during both the modeling and dosing phases. Following gavage, the mice were placed in separate, clean cages. 0.5 mL centrifuge tubes were used to hold the newly deposited faeces, and then the excrement was weighed to determine its wet mass. Samples underwent desiccation at 90 °C until weight stabilization. The water content in faeces was determined using Equation (1) [33]:

(1) $$Faeces\;water\;content\left(\%\right)=\frac{wet\;weight\;of\;the\;faeces\left(g\right)-dry\;weight\;of\;the\;faeces(g)}{wet\;weight\;of\;the\;faeces\;(g)}\times 100\%$$

### 2.3. The First Black Stool Defecation Time

In the course of modeling and administering the drug, a combination of the activated carbon (Shanghai Meryer Chemical Technology Co., Ltd., Shanghai, China) and appropriate medication was introduced via gavage, and the elapsed time between activated carbon administration and the first observation of black stool per mouse was noted as the first black stool defecation time [34].

### 2.4. Small Intestine Propulsion Rate

Following 20 days of uninterrupted treatment, all groups of mice underwent a 16 h fasting period. On the 21st day, the control group was given a mixture of physiological saline and activated carbon. The other groups were given a mixture of the respective drug and activated carbon and were then subsequently euthanized after 30 min. The complete small intestine was excised from the pylorus to the cecum, and its length was recorded as the “total length of the small intestine”. Additionally, the section from the pylorus to the terminal carbon-advancing edge was measured and referred to as the “length of the terminal carbon-advancing edge” [35]. The formula utilized for determining the rate of small bowel advancement was the following (2):

(2) $$Small\;intestine\;propulsion\;rate\left(\%\right)=\frac{distance\;propelled\;by\;the\;activated\;carbon(cm)}{total\;length\;of\;the\;small\;intestine(cm)}\times 100\%$$

### 2.5. Western Blot Analysis

Colon tissues were initially rinsed with PBS that had been pre-cooled and then lysed on ice using a chilled RIPA buffer (Beyotime Biotechnology, Shanghai, China) augmented with a protease inhibitor cocktail (MedChemexpress, Monmouth Junction, NJ, USA) for a duration of 30 min. Following this, the lysate underwent centrifugation at 12,000 rpm for 10 min, allowing for the collection of the supernatant. The BCA Protein Assay Kit (Beyotime Biotechnology, China) was employed to ascertain the protein concentration. To separate the total protein, SDS-PAGE was utilized, and then the proteins were transferred onto a PVDF membrane (Merckmillipore, Darmstadt, Germany). This membrane was then subjected to blocking with 5% skim milk for 1 h. Subsequently, it was incubated overnight at 4 °C with the following primary antibodies: AQP3 (Affinity, Newport Beach, CA, USA,1:500), AQP4 (Proteintech, Rosemont, IL, USA, 1:5000), NHE-3 (Affinity, 1:1000), CLC-2 (Affinity, 1:2000), Claudin-1 (Proteintech, 1:4000), ZO-1 (Affinity, 1:1000), Occludin-1 (Proteintech, 1:20,000), and GAPDH (Proteintech, 1:20,000). After the incubation period, the membrane underwent three 10 min washes with TBST at room temperature. Subsequently, it was incubated for an hour at room temperature with secondary antibody (YEASEN, Shanghai, China, 1:500). The strips were visualized using SuperKine™ ECL (Abbkine Biotechnology Co., Ltd., Atlanta, GA, USA) along with the Chemiluminescent Imaging System (Image Quant LAS 4000, General Electric, Boston, MA, USA).

### 2.6. ELISA

We obtained blood samples and spun them at 3000 rpm, 4 °C, for 20 min. The clarified liquid was retained for subsequent assays. 5-Hydroxytryptamine (5-HT), Vasoactive Intestinal (VIP), Motilin (MTL), and Gastrin (GAS) in sera were quantified using ELISA Kits (Quanzhou Ruixin Biotechnology Co., Ltd., Quanzhou, China), following the manufacturer’s instructions. Readings were obtained via microplate spectrophotometry, and cytokine concentrations were determined based on the standard curve.

### 2.7. H&E Staining

The colon samples were fixed, dehydrated, and embedded in paraffin. The tissues embedded in paraffin were sectioned into 3 µm slices. Prior to staining, the sections were deparaffinized and rehydrated. Hematoxylin–eosin (H&E) staining was performed using standard techniques. Eosin (ZSGB-BIO, Beijing, China) was utilized to stain the cytoplasm, while hematoxylin (ZSGB-BIO, China) was used for nuclear staining. All tissue sections were examined using an optical microscope (Olympus, Tokyo, Japan).

### 2.8. Fecal Microbiota Analysis

Fresh feces were collected 20 days after administration, placed in sterile tubes, and immediately stored at −80 °C. Total genomic DNA was extracted using the CTAB/SDS method [36]. The V4 region of the 16S rRNA gene was amplified with barcoded primers suitable for the Illumina platform. Combined samples underwent sequencing via the Illumina NovaSeq platform (NOVOGENE Company Limited, Beijing, China), yielding 250 bp paired-end reads [37]. The sequences were quality-filtered and trimmed using QIIME and UCHIME. For alpha diversity, we calculated the Chao1 index, Shannon index, Observed OTUs, and Simpson index. For beta diversity, we employed principal coordinate analysis (PCoA) based on the Bray–Curtis dissimilarity matrix, explaining how this method visualized differences in microbial community compositions between samples. Illumina MiSeq sequencing, followed by computational analysis, was performed by Novogene.

### 2.9. Fecal Metabolomic Analysis

Fresh feces were collected 20 days after administration, transferred to sterile vials, and then flash-frozen at −80 °C. Metabolite isolation, LC-MS/MS analysis, and quality control analysis were conducted on the colon content samples. Qualitative and quantitative analyses of the metabolites were performed through data preprocessing using CD software (version 3.3) and database searching, followed by metabolite data information analysis. Leveraging the KEGG database, we systematically annotated the identified metabolites, elucidated their associated metabolic pathways, and then conducted principal coordinate analysis (PCoA) to visually illustrate the disparities in the metabolite profiles among the samples. In univariate analysis, Student’s *t*-tests were employed to compute the *p*-values of metabolites between the two groups, thereby assessing the statistical significance of the differences. Fold-change (FC) values were calculated to quantify the magnitude of these differences. Ultimately, metabolites with VIP scores exceeding 1, *p*-values less than 0.05, and FC values ≥ 2 or ≤0.5 were designated as differentially expressed metabolites for further investigation. Additionally, sequence data underwent bioinformatic processing via the Illumina MiSeq system at Novogene.

### 2.10. Statistical Analysis

Data analysis was performed using GraphPad Prism 9.0 software (GraphPad Software, San Diego, CA, USA). To compare two groups, Student’s *t*-test was used. For comparisons involving more than two groups, one-way ANOVA was used. For multiple groups and two or more variables to consider, a two-way ANOVA was used. All results were shown as means ± SD. * *p* < 0.05 and ** *p* < 0.01 indicated significant differences.

## 3. Results

### 3.1. The Effect of L-Arabinose on Defecation-Related Parameters in Functionally Constipated Mice

**Figure 1:**
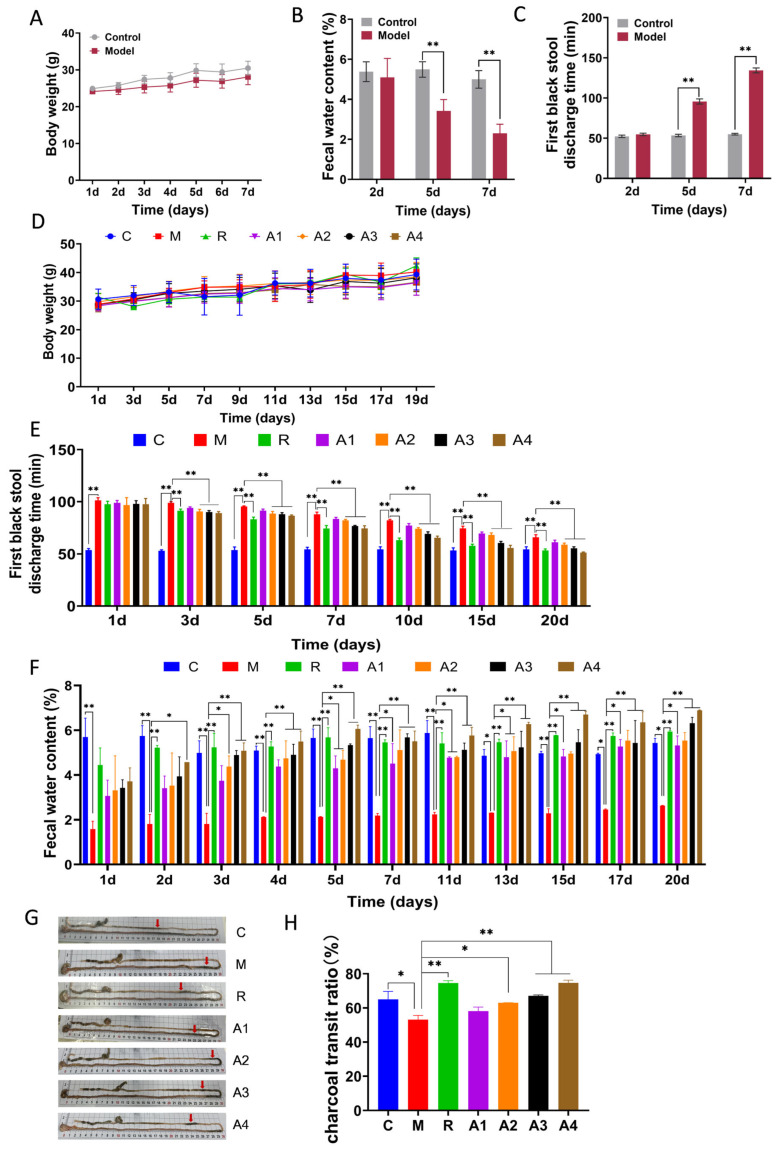
**Effect of L-Arabinose on defecation-related parameters in functionally constipated mice:** (**A**) body weight of mice during modeling; (**B**) fecal water content of mice during modeling; (**C**) the first black stool defecation time during modeling; (**D**) body weight of mice during administration; (**E**) the first black stool defecation time during administration; (**F**) water content of fecal water of mice during administration; (**G**) images of the small intestine propulsion rate of mice during administration; and (**H**) rate of the small intestine propulsion rate of mice during administration. * *p* < 0.05 and ** *p* < 0.01 indicate significant differences. C: control group; M: model group; R: lactulose group; A1: 0.5 g/kg L-Arabinose; A2: 0.75 g/kg, L-Arabinose; A3: 1.0 g/kg L-Arabinose; and A4: 2.0 g/kg L-Arabinose.

The constipated mouse model was induced using the compound diphenoxylate. After modeling, the mice exhibited a slight reduction in body weight compared to the control group (Figure 1A). Additionally, the mice in the model group showed a significantly lower fecal water content, and the first black stool defecation time took longer to appear (Figure 1B,C), thereby indicating successful modeling. As illustrated in Figure 1D, after L-Arabinose administration, the body weight of the mice in each group exhibited no significant variance. This indicated that L-Arabinose treatment did not have an adverse effect on the general physical condition of the mice during the experiment. Compared to the model group, the positive control group treated with lactulose demonstrated a reduction in the first black stool defecation time and an increase in the fecal water content (Figure 1E,F). Meanwhile, L-Arabinose treatment shortened the first black stool defecation time and increased the fecal water content in mice (Figure 1E,F). Compared to the model group, lactulose and different concentrations of L-Arabinose treatment enhanced the rate of activated carbon propulsion in the mice. Among them, several higher doses of L-Arabinose groups showed significant differences compared to the model group (Figure 1G,H). These findings suggest that L-Arabinose has the potential to ameliorate functional constipation.

### 3.2. The Effects of L-Arabinose on Colon Tissue Morphology, Functional Constipation-Related Intestinal Protein Expression, and Serum Neurotransmitter Levels

#### 3.2.1. The Effect of L-Arabinose on the Morphology of Colon Tissue in Functionally Constipated Mice

**Figure 2:**
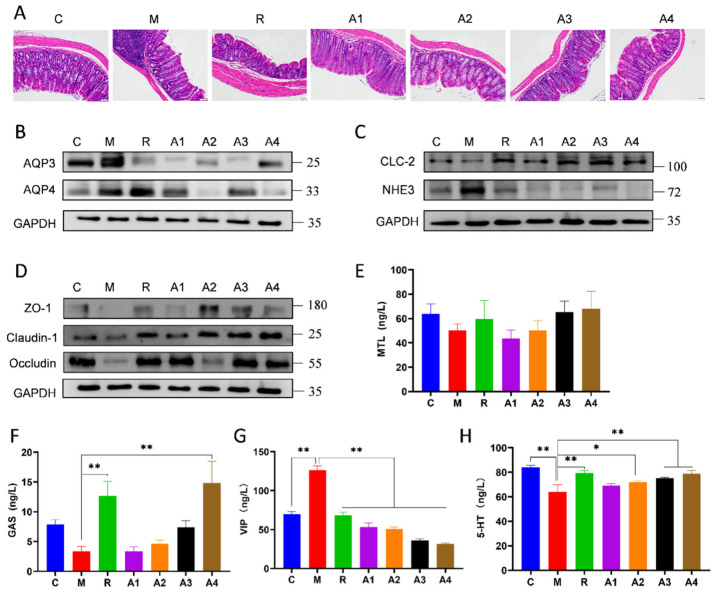
Effects of L-Arabinose on the histomorphology of colon, serum neurotransmitters, and expression levels of constipation-associated intestinal proteins: (**A**) histopathological analysis of the colon by H&E staining; (**B**) protein expression level of water channel proteins (AQP3 and AQP4); (**C**) protein expression level of ion channel proteins (NHE3 and CLC-2); (**D**) protein expression level of tight-junction proteins (ZO-1, Claudin-1, and Occludin); (**E**) serum MTL level in each group; (**F**) serum GAS level in each group; (**G**) serum VIP level in each group; and (**H**) serum 5-HT level in each group. * *p* < 0.05 and ** *p* < 0.01 indicate significant differences.

The observation of H&E-stained colon tissue sections (Figure 2A) revealed that the colonic tissues of all mouse groups exhibited structural integrity. In particular, the local mucosal lamina propria of the model group displayed a higher infiltration of inflammatory cells, mainly lymphocytes. Additionally, the intermuscular layer showed similar lymphocyte infiltration. Furthermore, the administration of L-Arabinose reduced the number of inflammatory cells, thereby mitigating the effects of inflammation.

#### 3.2.2. The Effect of L-Arabinose on the Expression Level of Functional Constipation-Related Intestinal Proteins

The abnormal expression of aquaporins (AQPs) may trigger imbalances in fluid absorption within the gut [38]. Therefore, the treatment of functional constipation may be achieved by regulating the levels of AQPs in the colonic mucosa. AQP3 and AQP4 expression increased in the experimental cohort relative to the controls, which might be associated with the disrupted water balance in the constipated state. L-Arabinose treatment reduced the expression levels of AQP3 and AQP4 to varying degrees (Figure 2B), indicating its potential role in restoring normal water balance. Additionally, the expression of the sodium hydrogen Na^+^/H^+^ exchanger 3 (NHE3) protein improved within the experimental group relative to the control, concurrent with a marked decline in chloride channel 2 (CLC2) protein levels. Following L-Arabinose intervention, the expression level of the NHE3 protein decreased, and the expression level of the CLC2 protein increased. The results indicate that L-Arabinose can influence aquaporins and ion channel proteins to regulate osmotic pressure and maintain intestinal water balance, thereby alleviating functional constipation (Figure 2C).

Tight-junction proteins are essential structural components that form the tight junctions of intestinal epithelial cells [39]. In contrast to the control group, the levels of ZO-1, Claudin-1, and Occludin in the model group were reduced, suggesting a potential impairment of the intestinal epithelial barrier in the functionally constipated model (Figure 2D). In contrast, the intervention employing L-Arabinose resulted in increased levels of all tight-junction proteins, suggesting that L-Arabinose may effectively repair the integrity of the intestinal epithelial barrier.

#### 3.2.3. The Effect of L-Arabinose on Hormones and Neurotransmitters in Functionally Constipated Mice

To investigate whether L-Arabinose could regulate intestinal function by influencing intestinal neurotransmitter content, hormones (MTL, Gas) and neurotransmitters (5-HT, VIP) were analyzed in the serum of constipated mice. Compared to the control group, the level of Gas and MTL in the serum of model mice was reduced, while it increased in the L-Arabinose treatment groups. Concurrently, the model group of mice exhibited a decrease in the 5-HT levels and an increase in the VIP levels. L-Arabinose intake was shown to elevate 5-HT levels and decrease VIP levels in constipated mice. Moreover, L-Arabinose regulated the aforementioned hormones and neurotransmitters in a dose-dependent manner (Figure 2E–H). This indicates that L-Arabinose has the potential to regulate hormone and neurotransmitter levels, thereby alleviating functional constipation in mice.

### 3.3. The Effect of L-Arabinose on Intestinal Microorganisms in Functionally Constipated Mice

#### 3.3.1. The Effect of L-Arabinose on the Abundance and Diversity of Gut Microbiota in Functionally Constipated Mice

**Figure 3:**
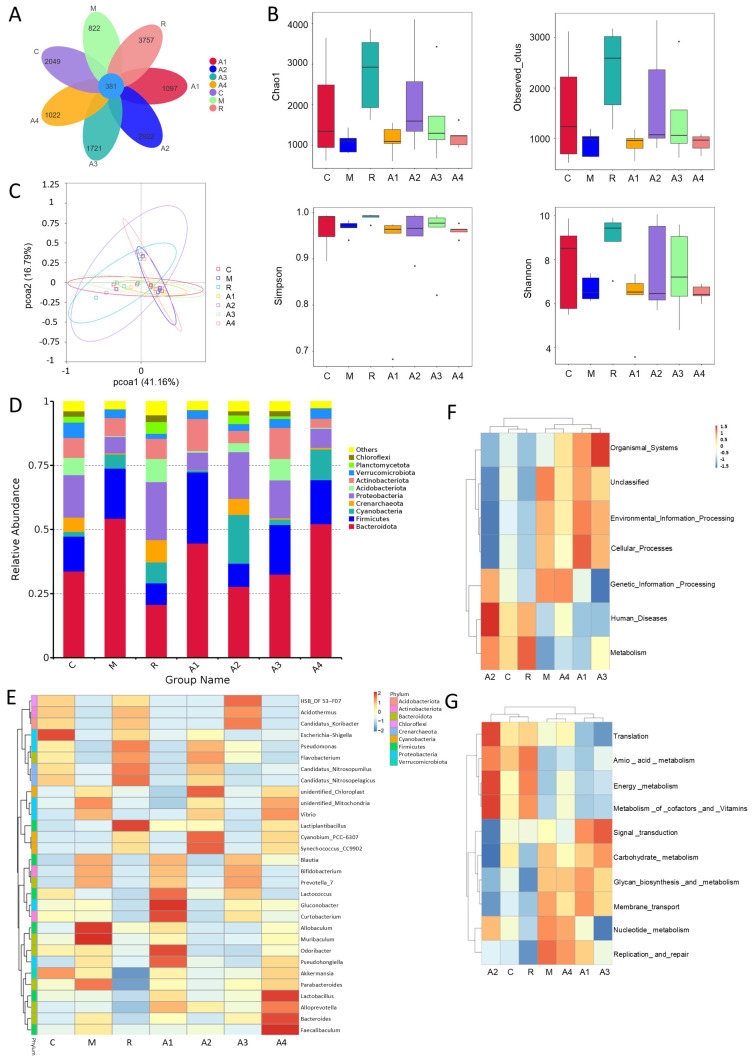
**Effect of L-Arabinose on gut microorganisms in functionally constipated mice:** (**A**) number of OTUs in each group; (**B**) alpha diversity; (**C**) principal coordinate analysis (PCoA); (**D**) variation in intestinal flora at the phylum level, according to Simpson; (**E**) heatmap of differences in intestinal flora at the genus level; (**F**) primary function prediction of 16S rRNA obtained using Tax4Fun; and (**G**) secondary function prediction of 16S rRNA obtained using Tax4Fun.

The number of colonic microbial operational taxonomic units (OTUs) in each group of mice is presented in Figure 3A. The specific OTU value in the control group was 2049, while that in the model group was 822, and the lactulose group exhibited an OTU number of 3757. The OTU values in the L-Arabinose groups were higher than those in the model group, especially the specific OTU number in group A2, which was higher than that in the control group. These findings indicate that functional constipation results in a reduced diversity of intestinal flora in mice, whereas consumption of L-Arabinose has the potential to boost this microbial abundance.

The alpha diversity in mouse feces is illustrated in Figure 3B. The Chao1 and Observed indices were utilized to assess colony abundance, while the Simpson and Shannon indices were employed to evaluate colony diversity. Compared to the control group, the model group showed reduced values across all metrics. The lactulose group demonstrated increases in all indices relative to the model group. Furthermore, in all dose groups of L-Arabinose, all indices, except for Simpson’s index, were restored compared to the model group.

The β-diversity analysis highlights varied gut microbiota across groups, with the model group deviating significantly from the controls (Figure 3C). Administration of L-Arabinose can restore β-diversity in mice, thus aiding in the recovery of intestinal flora homeostasis.

These findings indicate that the functional constipation model induced by compound diphenoxylate tablets decreases the abundance and diversity of colonic microorganisms; however, the intake of L-Arabinose enhances both the abundance and diversity of colonic microorganisms.

#### 3.3.2. Microbiota Variation

At the phylum level of the intestinal flora of the groups in our study (Figure 3D), we found that the primary differences were in the relative abundance of four key microbial phyla: Firmicutes, Bacteroidetes, Proteobacteria, and Acidobacteriota. The data reveal that, compared to the control group, the model group had a higher abundance of Bacteroidetes and a lower abundance of Acidobacteriota and Proteobacteria. This alteration might have been related to the disrupted intestinal environment caused by functional constipation. After L-Arabinose administration, the abundance of Bacteroidetes decreased across all groups, except for the A4 group. L-Arabinose administration was also effective in elevating the abundance of Acidobacteriota and Proteobacteria, indicating its positive role in restoring the balance of the intestinal flora. The specific numerical changes in phylum-level microbiota are shown in Table S1.

Further analysis at the genus level of the intestinal flora (Figure 3E) revealed that the top 30 microorganisms identified in all groups of mice included genera such as *Lactobacillus*, *Bacteroides*, *Akkermansia*, and *Allobaculum*, among others. These diverse genera play crucial and distinct roles in maintaining gut health. Specifically, *Lactobacillus* enhances intestinal mucosal barrier function, reduces harmful substance stimulation, and lowers constipation risk. *Lactococcus* repairs the gut flora barrier and treats intestinal diseases. *Acidothermus* stimulates the immune system, boosts immunity, and prevents infections and diseases. In contrast, commonly present in the gut, *Vibrio* can cause intestinal infections like diarrhea. These functions suggest that a balanced gut microbiota is essential for preventing constipation. Against this backdrop, compared to the control group, mice in the model group showed a decrease in the number of beneficial microorganisms, such as *Lactococcus* and *Acidothermus*. Meanwhile, there was an increase in the number of harmful microorganisms, such as *Vibrio*. This imbalance in the gut microbiota is likely a contributing factor to the development of functional constipation. Given this imbalance, we explored the potential of L-Arabinose intervention. We found that L-Arabinose intervention modulates these changes in microbial composition and the abundance associated with functional constipation by increasing the levels of beneficial bacteria such as *Lactobacillus*, *Acidothermus*, and *Lactococcus*, while decreasing the levels of harmful bacteria such as *Bacteroides*, *Vibrio*, and *Pseudohongiella*. In this way, L-Arabinose helps to restore the balance of the gut microbiota, which may effectively alleviate the symptoms of functional constipation. The specific numerical changes in genus-level microbiota are shown in Table S2.

#### 3.3.3. Prediction of Microbial Function

This study utilized Tax4Fun software (version V0.3.1) to annotate the KEGG pathway of the colonic flora based on the OTU abundance information [40]. As illustrated in Figure 3F, the KEGG pathway predicted by the gut microbiota in each group of mice included six typical pathways—metabolism, genetic information processing, environmental information processing, cellular processes, human diseases, and organismal systems. Further secondary prediction of metabolite function revealed that the model group was down-regulated at the level of the amino acid metabolism and energy metabolism pathways compared to the control group and that the administration of the L-Arabinose A2 group and lactulose enhanced said pathways (Figure 3G).

### 3.4. The Effect of L-Arabinose on Intestinal Metabolites in Functionally Constipated Mice

**Figure 4:**
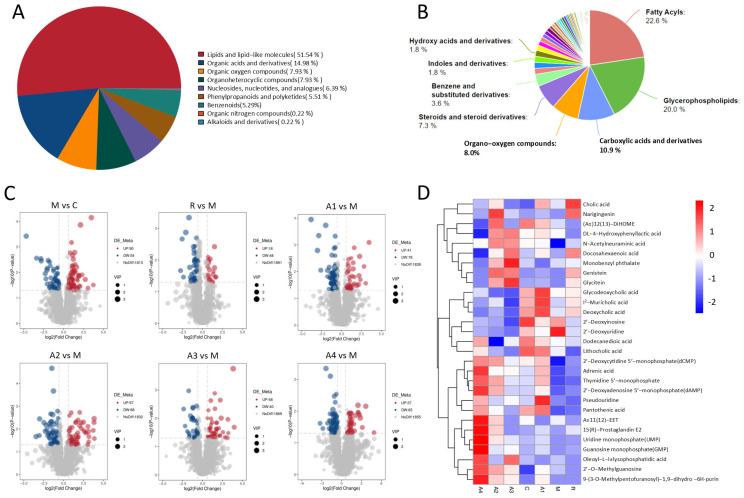
**Effect of L-Arabinose on intestinal metabolites in functionally constipated mice:** (**A**) classification of primary metabolites; (**B**) secondary metabolites’ classification; (**C**) differential metabolite volcano plot; and (**D**) cluster analysis of total differential metabolites.

As illustrated in Figure 4A, the classification of metabolites predominantly comprised lipid and lipid-like molecules (51.54%), organic acids and derivatives (14.98%), organoheterocyclic compounds (7.93%), and organic oxygen compounds (7.93%). Further categorization of these metabolites yielded a Class II classification pie chart, as shown in Figure 4B. This chart indicates that the primary classifications were fatty acyls (22.6%), glycerophospholipids (20.0%), carboxylic acids and derivatives (10.9%), and organo-oxygen compounds (8.0%).

In terms of the differences in metabolites between the two groups and within the whole group (Figure 4C,D), the key fecal metabolites associated with constipation were muricholic acid, deoxycholic acid, lithocholic acid, dodecanedioic acid, pantothenic acid, and deoxyadenosine monophosphate. Compared to the control group, functional constipation resulted in the down-regulation of colonic metabolites, including muricholic acid, deoxycholic acid, lithocholic acid, dodecanedioic acid, and pantothenic acid. Conversely, the administration of L-Arabinose elevated the levels of these metabolites, namely muricholic acid, deoxycholic acid, lithocholic acid, dodecanedioic acid, and pantothenic acid. Muricholic acid, deoxycholic acid, and lithocholic acid are bile acids. Physiologically, bile acids in the intestinal cavity stimulate colon movement. Besides stimulating colon movement, bile acids can also relieve constipation by altering the intestinal flora structure [41,42]. Dodecanedioic acid increases the intestinal water content, softening stools [43]. Pantothenic acid promotes intestinal epithelial cell growth and repair, strengthens the mucosal barrier, and improves constipation symptoms [44]. This regulation of related metabolites helps to further balance intestinal bacterial homeostasis, thereby maintaining intestinal health.

### 3.5. The Effect of L-Arabinose on Intestinal Metabolite Pathways in Functionally Constipated Mice

**Figure 5:**
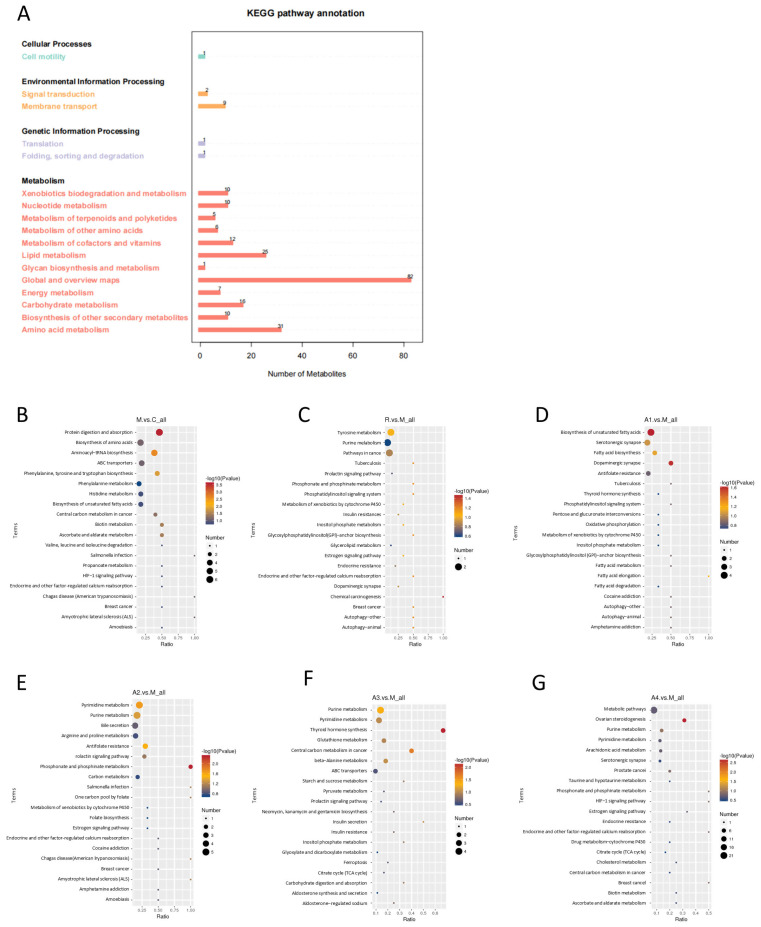
**Effect of L-Arabinose on intestinal metabolite differential KEGG pathway in functionally constipated mice:** (**A**) total differential metabolite KEGG pathway; and (**B**–**G**) KEGG pathway enrichment of DEGs in M vs. C group, R vs. M group, A1 vs. M group, A2 vs. M group, A3 vs. M group, and A4 vs. M group.

A total of 229 metabolic pathways are annotated in the metabolic pathway depicted in Figure 5A. The primary metabolic pathways encompass metabolism, genetic information processing, environmental information processing, and cellular processes. A comprehensive analysis of these primary metabolic pathways reveals secondary pathways related to functional constipation, including carbohydrate metabolism, amino acid metabolism, lipid metabolism, and nucleotide metabolism. Among these, amino acid metabolism, lipid metabolism, and carbohydrate metabolism emerge as the major regulatory pathways. Further investigation into the metabolic pathways within the colonic segments of mice in each group, as illustrated in Figure 5B–G, revealed that the primary metabolic pathways L-Arabinose affected included lipid metabolism, amino acid metabolism, and nucleotide metabolism.

### 3.6. Association Analysis of Gut Microorganisms and Metabolites in Constipated Mice

**Figure 6:**
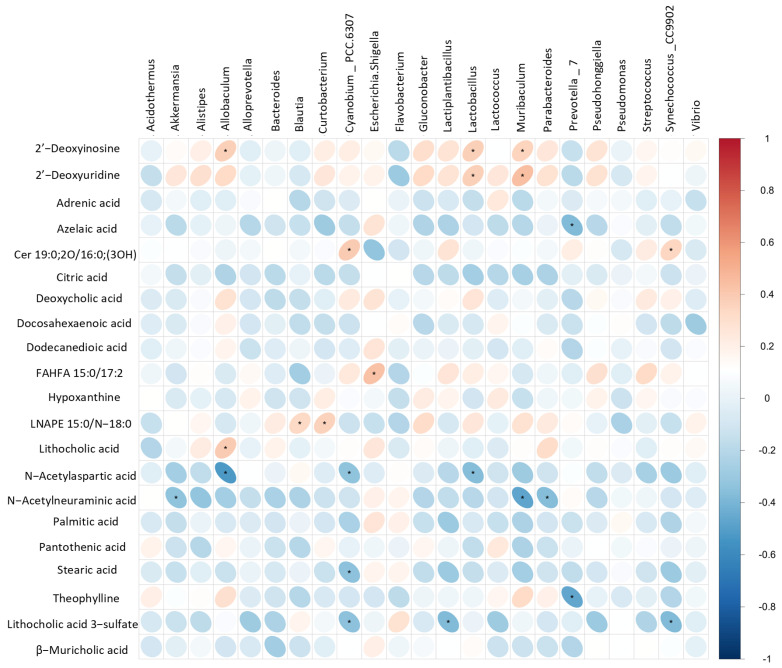
**Heatmap analysis of Spearman correlations between differential metabolites and dominant genera.** Negatively sloped ellipses indicate negative correlations, and positively sloped ellipses indicate positive correlations. * *p* < 0.05 indicate significant differences.

Further, we assessed the Spearman correlation coefficients between differential metabolites and gut bacterial abundance to explore the functional relationship between altered gut flora and changes in fecal metabolites (Figure 6). The results indicated that the levels of numerous fecal metabolites exhibited strong correlations with the relative abundance of dominant microbial genera. Significantly, lithocholic acid levels showed a positive correlation with the abundance of *Allobaculum*, which belongs to the phylum Bacteroidetes. The increase in Allobaculum levels suggests a potential link to increased lithocholic acid biosynthesis, which aligns with previous reports indicating that lithocholic acid-producing bacteria predominantly belong to the phylum Bacteroidetes. Additionally, the metabolites deoxycholic acid, pantothenic acid, and muricholic acid showed negative correlations with *Vibrio*, while deoxycholic acid was positively correlated with *Lactococcus*. Overall, L-Arabinose enhances the intestinal microecological environment and promotes intestinal health by influencing various dominant flora (e.g., Lactobacillus, *Alloprevotella*, and *Bacteroides*) and their metabolites (e.g., stearic acid, pantothenic acid, deoxycholic acid, and dodecanedioic acid). These changes encompass the enhancement of intestinal barrier function, the regulation of lipid and glucose metabolism, the promotion of nutrient absorption, the reduction in functional constipation risk, the maintenance of intestinal homeostasis, and the mitigation of potential intestinal damage from harmful substances.

## 4. Discussion

Functional constipation, a common gastrointestinal disorder, involves slowed intestinal motility and defecatory challenges [45]. The significant side effects associated with commonly used medications, such as laxatives, like abdominal cramps, electrolyte imbalances, and dependency issues, underscore the need for alternative interventions [46]. There are reports of compound diphenoxylate tablet treatment being able to establish a functional constipation mouse model [47]. Hower, while numerous studies have indicated that prebiotics or polysaccharides are effective in alleviating functional constipation [48,49], there is a paucity of research focusing on the efficacy of L-Arabinose in this regard. In this study, we analyzed the alleviating effect of L-Arabinose on functionally constipated mice and explained its mechanism for promoting excretion.

Functionally constipated mice frequently exhibit impaired gastrointestinal motility [50], which may be reflected in the levels of gastrointestinal active peptides [51]. These peptides can be categorized into two groups: excitatory and inhibitory [52]. The distinction between these two types is crucial as they work in tandem to precisely regulate the complex processes of gastrointestinal motility, with excitatory peptides generally promoting and inhibitory peptides typically modulating or suppressing certain aspects of the gut’s movement. In this study, four types of gastrointestinal peptides were examined: MTL, GAS, VIP, and 5-HT. MTL, GAS, and 5-HT are classified as excitatory peptides, while VIP is inhibitory. MTL stimulates the production of pepsin and promotes duodenal contraction [53]. The regulation of gastrointestinal active peptides promotes digestive function in the digestive tract, which can lead to constipation when disturbed. GAS facilitates pyloric sphincter relaxation and the contraction of gastrointestinal smooth muscle [54]. This relaxation effect is part of the complex regulatory mechanism within the gastrointestinal tract, as it helps to counterbalance the contractions induced by excitatory peptides and maintain an appropriate level of muscle tone, which is necessary for normal bowel movements and can impact the severity of functional constipation symptoms. VIP plays a role in relaxing smooth muscle, both contributing to gastrointestinal motility. The proper functioning of the pyloric sphincter and smooth muscle contractions are vital for the coordinated movement of food through the digestive system, and any imbalance in these actions could potentially lead to functional constipation-related issues [55]. 5-HT modulates gastrointestinal motility primarily by stimulating the enteric nervous system (ENS), which subsequently enhances the secretion of gastrointestinal mucus [34], dilates blood vessels, and contracts smooth muscle. The combined effects of mucus secretion, blood vessel dilation, and smooth muscle contraction under the influence of 5-HT are integral to the proper functioning of the gastrointestinal tract. Any alteration in 5-HT levels can thus affect the overall motility and potentially contribute to functional constipation or its alleviation. In this study, L-Arabinose was found to restore normal serum levels of two key types of neurotransmitters: excitatory neurotransmitters such as GAS, MTL, and 5-HT, and inhibitory neurotransmitters like VIP. As a result of this modulation, it optimized peristalsis in the intestinal tract, thereby promoting smooth defecation and alleviating symptoms of functional constipation.

The abnormal expression of aquaporins (AQPs) in the colon can lead to excessive water absorption in the intestine or reduced intestinal fluid secretion [56], which may contribute to functional constipation. Specifically, AQP3 and AQP4 are predominantly expressed in mucosal epithelial cells and play crucial roles in intestinal water reabsorption [57]. Research has indicated that alleviating functional constipation may be achieved by decreasing the levels of AQP3 and AQP4 [58]. CLC2 and NHE3 are essential ion transporters that significantly contribute to the body’s regulation of water and electrolyte balance by controlling ion movement across cell membranes. In this study, L-Arabinose not only reduced the levels of water channel proteins AQP3, AQP4, and NHE3 but also increased the levels of the CLC2 protein. This alteration aids in minimizing unnecessary water loss from the intestinal tract, maintaining a moist intestinal environment, and ultimately facilitating softer and more easily passable feces. Furthermore, tight-junction proteins are crucial structural components that form the tight junctions of intestinal epithelial cells, playing a vital role in regulating trans-epithelial transport, cell proliferation, and differentiation. Enhancing the levels of tight-junction proteins such as ZO-1, Occludin, and Claudin-1 can further ameliorate functional constipation by repairing the intestinal barrier [59].

Functional constipation is a prevalent disorder that is closely associated with the intestinal flora [60]. Research indicates that dietary fiber [38] and polysaccharides can alleviate constipation by enhancing the population of beneficial bacteria in the intestines while reducing harmful bacteria [49]. In the present study, L-Arabinose notably enhanced gut microbiota abundance and the diversity of the intestinal flora. L-Arabinose can regulate the levels of abnormal Bacteroidetes, Firmicutes, and Proteobacteria species and improve the composition of intestinal bacteria. Genus-specific analysis showed that, compared to the control group, the level of *Lactobacillus* was reduced and the level of *Bacteroides* was elevated in the model group. However, L-Arabinose application reversed this trend.

Intestinal microbe metabolites are frequently linked to constipation development [61,62]. For example, Lactulose eases constipation and increases the synthesis of favorable metabolites. Notably, it can alleviate constipation by enhancing the production of beneficial metabolites, including bile acids and short-chain fatty acids. Additionally, konjac glucomannan has been shown to influence the levels of amino acids, choline, and indole-phenol sulfate, contributing to the relief of constipation, among other effects [63,64]. In this study, we observed that functional constipation is associated with the down-regulation of several colonic metabolites, including deoxycholic acid, lithocholic acid, dodecanedioic acid, and pantothenic acid. Conversely, L-Arabinose treatment elevated the levels of metabolites and stimulated gut motility. This intervention also maintained the balance of intestinal bacterial homeostasis, thereby alleviating functional constipation and promoting overall intestinal health.

This study suggests that L-Arabinose may have an impact on the intestinal microecological environment. It was observed that there were changes in several dominant microbial populations, like *Lactobacillus*, *Acidothermus*, and *Bacteroides*, along with alterations in their metabolites, such as lithocholic acid, pantothenic acid, deoxycholic acid, and dodecanedioic acid. However, the relationship between these observed changes and the overall promotion of intestinal health in humans remains to be further explored and established.

Compared with other prebiotics, L-Arabinose not only exhibits unique advantages in physiological functions and source characteristics but also has distinct features from the perspective of relevant research. In terms of physiological functions, it can specifically inhibit sucrose absorption, conferring dual health benefits by regulating blood glucose levels and alleviating constipation. As a common component of various plant polysaccharide hemicelluloses, L-Arabinose has a wide range of sources and diverse production methods. In the research field of constipation alleviation, most prebiotics often focus on a single function. We delved deep into the interactions between gut microbiota and metabolite profiling, as well as feedback regulation via intestinal-related key proteins, to analyze the effect of L-Arabinose on functional constipation. As studies have shown, L-Arabinose can precisely regulate key microbial communities in the colon and modulate the metabolic pathways of key metabolites, thereby relieving constipation. Therefore, L-Arabinose has potential as a new food resource component for preventing functional constipation.

## 5. Conclusions

Our study demonstrates that the mechanism of action of L-Arabinose for alleviating functional constipation may involve the precise modulation of dominant microbial communities, including *Lactobacillus*, *Bacteroides*, and *Lactococcus*, within the colonic microecosystem. Additionally, it appears to influence the metabolic pathways of critical metabolites such as muricholic acid, deoxycholic acid, and lithocholic acid. This modulation initiates a series of complex biochemical cascades that significantly impact the levels of VIP, 5-HT, MTL, and Gas, all of which are essential biochemical signaling molecules in the regulation of gastrointestinal tract functions. Further analysis indicates that L-Arabinose may enhance the production of beneficial metabolites, such as stearic acid and palmitic acid, through lipid metabolism, amino acid metabolism, and nucleotide metabolism, while simultaneously inhibiting the formation of potentially harmful metabolites like trimethylamine. These alterations operate at the cellular level, influencing the expression and functionality of key proteins directly associated with functional constipation symptoms, such as AQP3, AQP4, CLC-2, and NHE3. This further regulates the balance of intestinal water absorption and secretion, promoting the softening and smooth passage of intestinal contents.

By integrating multiple mechanisms, L-Arabinose alleviates functional constipation symptoms. Initially, it boosts probiotic proliferation and metabolite synthesis, which helps to improve the intestinal microecological environment. Secondly, it enhances gastrointestinal motility, enabling the smooth movement of intestinal contents. Thirdly, it regulates intestinal osmotic pressure, which further aids in the softening and passage of feces. Fourthly, it strengthens the intestinal barrier, preventing the entry of harmful substances into the intestine. Finally, it restores the levels of neurotransmitters, which are essential for the regulation of gastrointestinal functions. All these effects indicate that L-Arabinose has promising potential as a new food resource ingredient for preventing functional constipation.

## Data Availability

The data presented in this study are available on request from the corresponding author. The data are not publicly available due to privacy restrictions.

## Supplementary Materials

The following supporting information can be downloaded at https://www.mdpi.com/article/10.3390/foods14050900/s1: Table S1: Analysis of the gut microbiota at the phylum level in functional constipation model mice during administration; and Table S2: Analysis of the gut microbiota at the genus level in functional constipation model mice during administration.
