# Supplementary material for: L-Arabinose Alleviates Functional Constipation in Mice by Regulating Gut Microbiota and Metabolites
Source: Foods. 2025 Mar 6;14(5):900. doi: 10.3390/foods14050900 (PMC11899279; doi:10.3390/foods14050900)
Supplement: Supplementary file 1 [file foods-14-00900-s001.zip › foods-3494943-supplementary.pdf]

Table S1. Analysis of the gut microbiota at the phylum level in functional constipation model mice during administration.

| category | C | M | R | A1 | A2 | A3 | A4 |
|---|---|---|---|---|---|---|---|
| Bacteroidota | 3.38E-01 | 5.42E-01 | 2.07E-01 | 4.46E-01 | 2.78E-01 | 3.26E-01 | 5.22E-01 |
| Firmicutes | 1.35E-01 | 1.96E-01 | 8.37E-02 | 2.78E-01 | 9.09E-02 | 1.92E-01 | 1.72E-01 |
| Cyanobacteria | 1.84E-02 | 5.45E-02 | 8.20E-02 | 7.10E-03 | 1.89E-01 | 2.14E-02 | 1.20E-01 |
| Crenarchaeota | 5.64E-02 | 4.90E-03 | 8.65E-02 | 1.40E-03 | 6.27E-02 | 5.00E-03 | 6.40E-03 |
| Proteobacteria | 1.65E-01 | 6.41E-02 | 2.26E-01 | 6.80E-02 | 1.83E-01 | 1.48E-01 | 7.47E-02 |
| Acidobacteriota | 6.71E-02 | 3.10E-03 | 9.00E-02 | 6.60E-03 | 3.51E-02 | 8.36E-02 | 3.60E-03 |
| Actinobacteriota | 7.78E-02 | 7.02E-02 | 7.87E-02 | 1.26E-01 | 4.81E-02 | 1.21E-01 | 3.55E-02 |
| Verrucomicrobiota | 6.04E-02 | 3.36E-02 | 1.96E-02 | 3.29E-02 | 2.63E-02 | 3.54E-02 | 3.93E-02 |
| Planctomycetota | 2.30E-02 | 2.00E-04 | 4.56E-02 | 2.00E-04 | 3.32E-02 | 1.00E-02 | 1.00E-03 |
| Chloroflexi | 2.07E-02 | 1.00E-03 | 2.64E-02 | 1.10E-03 | 1.66E-02 | 2.00E-02 | 1.00E-03 |
| Others | 3.76E-02 | 2.94E-02 | 5.31E-02 | 3.29E-02 | 3.75E-02 | 3.73E-02 | 2.56E-02 |

Table S2. Analysis of the gut microbiota at the genus level in functional constipation model mice during administration.

| category | C | M | R | A1 | A2 | A3 | A4 |
|---|---|---|---|---|---|---|---|
| Lactococcus | 6.37E-02 | 1.95E-02 | 5.30E-03 | 1.11E-01 | 2.98E-02 | 8.28E-02 | 8.80E-03 |
| Cyanobium_PCC-6307 | 0.00E+00 | 0.00E+00 | 5.11E-02 | 0.00E+00 | 8.62E-02 | 0.00E+00 | 5.30E-02 |
| unidentified_Chloroplast | 1.82E-02 | 5.30E-02 | 2.25E-02 | 5.70E-03 | 8.80E-02 | 2.11E-02 | 5.80E-02 |
| Akkermansia | 4.61E-02 | 3.31E-02 | 2.90E-03 | 3.21E-02 | 1.99E-02 | 2.23E-02 | 3.88E-02 |
| Alloprevotella | 2.49E-02 | 2.99E-02 | 1.22E-02 | 6.87E-02 | 5.22E-02 | 3.61E-02 | 8.10E-02 |
| Bacteroides | 2.58E-02 | 5.61E-02 | 1.83E-02 | 4.53E-02 | 2.33E-02 | 5.07E-02 | 9.52E-02 |
| Lactiplantibacillus | 1.40E-03 | 2.70E-03 | 2.43E-02 | 8.10E-03 | 8.80E-03 | 1.00E-03 | 1.26E-02 |
| Pseudomonas | 2.80E-02 | 1.70E-03 | 4.88E-02 | 3.10E-03 | 4.06E-02 | 1.85E-02 | 4.30E-03 |
| Candidatus_Nitrosopumilus | 1.38E-02 | 2.50E-03 | 2.11E-02 | 4.00E-04 | 1.65E-02 | 1.00E-04 | 3.10E-03 |
| Bifidobacterium | 1.00E-04 | 1.91E-02 | 2.00E-04 | 1.77E-02 | 1.00E-04 | 1.95E-02 | 3.00E-04 |
| unidentified_Mitochondria | 4.60E-03 | 1.81E-02 | 4.30E-03 | 6.00E-04 | 1.21E-02 | 4.30E-03 | 1.68E-02 |
| Lactobacillus | 5.00E-03 | 4.70E-03 | 1.50E-03 | 1.08E-02 | 3.80E-03 | 5.80E-03 | 1.82E-02 |
| Synechococcus_CC9902 | 0.00E+00 | 0.00E+00 | 7.90E-03 | 0.00E+00 | 1.37E-02 | 0.00E+00 | 7.80E-03 |
| Prevotella_7 | 2.00E-04 | 1.15E-02 | 9.00E-04 | 1.02E-02 | 1.50E-03 | 1.21E-02 | 1.00E-03 |
| Curtobacterium | 7.40E-03 | 5.60E-03 | 6.00E-04 | 1.92E-02 | 8.00E-04 | 1.07E-02 | 4.00E-03 |
| Faecalibaculum | 9.00E-04 | 5.00E-03 | 8.00E-04 | 1.10E-03 | 2.00E-03 | 4.00E-04 | 1.19E-02 |
| Candidatus_Koribacter | 6.70E-03 | 1.00E-04 | 7.50E-03 | 5.00E-04 | 5.00E-04 | 9.80E-03 | 3.00E-04 |
| Vibrio | 1.00E-04 | 7.70E-03 | 1.00E-04 | 4.00E-04 | 6.80E-03 | 1.00E-04 | 8.80E-03 |
| HSB_OF53-F07 | 5.50E-03 | 1.00E-04 | 4.90E-03 | 2.00E-04 | 3.00E-04 | 8.50E-03 | 2.00E-04 |
| Allobaculum | 5.00E-04 | 8.80E-03 | 1.90E-03 | 4.30E-03 | 1.00E-04 | 0.00E+00 | 2.40E-03 |
| Flavobacterium | 7.00E-03 | 2.00E-04 | 1.14E-02 | 3.00E-04 | 1.11E-02 | 4.30E-03 | 1.00E-04 |
| Odoribacter | 5.30E-03 | 5.80E-03 | 2.90E-03 | 9.70E-03 | 2.00E-03 | 1.70E-03 | 4.00E-03 |
| Pseudohongiell | 1.00E-04 | 3.70E-03 | 6.00E-04 | 6.80E-03 | 1.10E-03 | 1.00E-04 | 4.30E-03 |
| Gluconobacter | 3.50E-03 | 4.20E-03 | 2.00E-04 | 1.38E-02 | 5.00E-04 | 5.30E-03 | 3.80E-03 |
| Muribaculum | 7.80E-03 | 1.92E-02 | 6.60E-03 | 1.00E-02 | 5.10E-03 | 5.80E-03 | 9.80E-03 |
| Escherichia-Shigella | 6.80E-03 | 7.00E-04 | 4.10E-03 | 1.00E-03 | 2.70E-03 | 5.00E-04 | 1.10E-03 |
| Acidothermus | 5.70E-03 | 4.00E-04 | 6.10E-03 | 7.00E-04 | 7.00E-04 | 7.10E-03 | 4.00E-04 |
| Parabacteroides | 9.10E-03 | 1.64E-02 | 3.00E-03 | 1.04E-02 | 7.60E-03 | 1.02E-02 | 1.32E-02 |

| | | | | | | | |
|---|---|---|---|---|---|---|---|
| Blautia | 2.00E-04 | 6.60E-03 | 4.00E-04 | 6.40E-03 | 3.00E-04 | 6.10E-03 | 2.60E-03 |
| Candidatus_Nitrosopelagicus | 3.90E-03 | 5.00E-04 | 5.70E-03 | 1.00E-04 | 3.70E-03 | 1.00E-04 | 3.00E-04 |
